# Optical manipulation of molecular function by chromophore-assisted light inactivation

**DOI:** 10.2183/pjab.97.011

**Published:** 2021-04-09

**Authors:** Kiwamu TAKEMOTO

**Affiliations:** *1 Department of Biochemistry, Mie University, Graduate School of Medicine, Tsu-City, Mie, Japan.

**Keywords:** chromophore-assisted light inactivation, optical manipulation

## Abstract

In addition to simple on/off switches for molecular activity, spatiotemporal dynamics are also thought to be important for the regulation of cellular function. However, their physiological significance and *in vivo* importance remain largely unknown. Fluorescence imaging technology is a powerful technique that can reveal the spatiotemporal dynamics of molecular activity. In addition, because imaging detects the correlations between molecular activity and biological phenomena, the technique of molecular manipulation is also important to analyze causal relationships. Recent advances in optical manipulation techniques that artificially perturb molecules and cells via light can address this issue to elucidate the causality between manipulated target and its physiological function. The use of light enables the manipulation of molecular activity in microspaces, such as organelles and nerve spines. In this review, we describe the chromophore-assisted light inactivation method, which is an optical manipulation technique that has been attracting attention in recent years.

## 1 Introduction

Recent green fluorescent protein (GFP) technology has shown the spatio-temporal dynamics of protein activation *in vitro* and *in vivo*. For example, Cdc42, one of a family of Rho GTPases, is rapidly activated and its molecular activity is confined to a single synapse during synaptic plasticity.^1)^ In addition, caspase, a cell death protease, is locally activated in anterior cells early in the programmed cell death of salivary glands in *Drosophila*.^2)^ These results were observed using GFP-based Förster resonance energy transfer analysis in living tissues and animals. Such local and temporal activation of molecules is observed in many other organisms, such as plants, where ROP11 GTPases are locally activated during pattern formation of the cell wall, which were highlighted by GFP-tagging.^3)^ However, to what extent are these spatiotemporal dynamics of molecular activity present in living tissue? Moreover, many relevant details, such as the physiological significance and contribution of these spatiotemporal dynamics *in vivo*, are largely unknown. One reason for this is that current technology lacks the ability to manipulate molecules instantaneously and locally. This technology is suggested to be the key to elucidating the causal relationship between the spatiotemporal behavior of molecules and their physiological functions. This review examines the potential of chromophore-assisted light inactivation (CALI) as an optical technique to address this issue.

**Figure 1.**
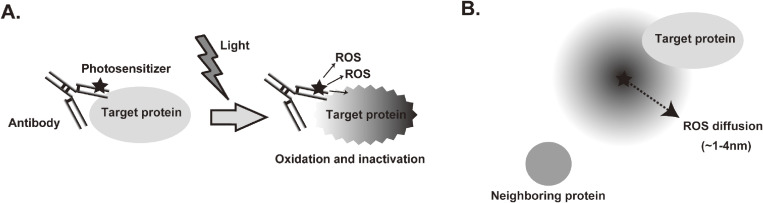
Schematic representation of chromophore-assisted light inactivation (CALI). **A.** In the CALI experiment, the antibody specific for the target protein is labeled with a photosensitizer. After irradiation with light, reactive oxygen species (ROS) are generated and oxidize the target protein. This destroys the structure of the target protein, resulting in inactivation. **B.** The half radius of ROS is an important factor for the efficiency and specificity of CALI.

CALI is a promising technique to induce the localized and acute inactivation of proteins by light.^4)^ The key compound of CALI is a photosensitizer that generates reactive oxygen species (ROS) in response to light irradiation. For example, antibodies for target proteins are chemically labeled with a photosensitizer. After the reaction of a labeled antibody with the target, light is administered to generate ROS from photosensitizers. When ROS attack the target protein in the local environment, the target protein should be inactivated via oxidation (Fig. 1A). Because the diffusion radius of ROS is very short (*e.g.*, singlet oxygen: 3–4 nm),^5)^ a specific target can be inactivated. Therefore, the efficiency and specificity of CALI depend on the short diffusion radius of ROS, suggesting that CALI efficiency should depend on the relative positional relationship between the photosensitizer and the target molecule, such as the distance and orientation (Fig. 1B). For an effective CALI experiment, it is important to place a photosensitizer in the vicinity of the target molecule, either by labeling specific antibodies with a chemical photosensitizer or by fusing and expressing a genetically encoded photosensitizing protein with the target molecule. The former method is difficult to apply to intracellular molecules but has the advantage of being able to target endogenous membrane surface molecules. The latter does not require antibodies and therefore allows for easy CALI experiments but requires gene knock-in of photosensitizing proteins for application to endogenous molecules. In any case, because it is difficult to theoretically control the relative positional relationship, the efficiency and specificity of CALI should be confirmed in individual experiments, for example, by using negative control experiments for neighboring proteins of the CALI target, the mutant cells and their rescue experiments.

## 2 Mechanisms of ROS generation by CALI

**Figure 2.**
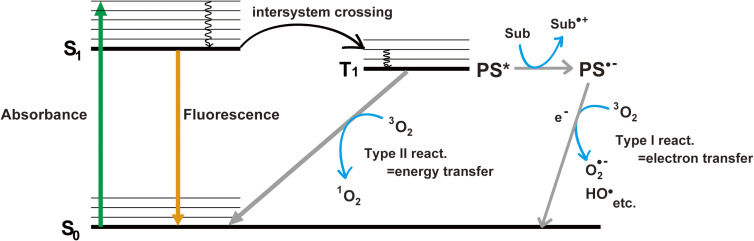
(Color online) The principles of reactive oxygen species (ROS) generation. In the Type I reaction, radicals are generated by electron transfer between the T_1_ photosensitizer (PS*) and the target molecule (Sub). In this figure, the generated photosensitizer radical anion (PS^•−^) then transfers an electron to oxygen to produce ROS such as superoxide radicals (O_2_^•−^) and hydroxyl radicals (HO^•^).^84)^ Note the opposite may also occur, depending on the redox potential of the pair of these molecules. In addition, in principle, the partner molecule for the electron exchange could be other than the target molecule.^8)^ In contrast, the Type II reaction directly produces singlet oxygen (^1^O_2_) by energy transfer from the T_1_ PS* to an oxygen.^6)^

There are two known mechanisms by which photosensitizers produce ROS after absorbing light: Type I and Type II reactions (Fig. 2).^6,7)^ In both reactions, it is important that the photosensitizer first absorbs light to enter the excited singlet state (S_1_) and then transitions to the excited triplet state (T_1_) through intersystem crossing. In the Type I reaction, radicals are generated as a result of electron transfer between the T_1_ photosensitizer (PS*) and the target molecule (Sub). The generated anion radicals (PS^•−^) then transfer an electron to ground state oxygen to produce ROS such as superoxide radicals (O_2_^•−^) and hydroxyl radicals (HO^•^). Whether PS* or Sub becomes an anion or cation in the first electron transfer reaction depends on the redox potential.^6)^ In addition, it should be noted that electron transfer occurs at a distance of ∼15 Å,^8)^ so in principle, the exchange of electrons may not have happened in the target molecule but in another nearby molecule. In contrast, the Type II reaction does not produce radicals but rather produces singlet oxygen (^1^O_2_) by energy transfer from the T_1_ PS* to a ground state oxygen. It has been reported that the diffusion radii of hydroxyl radical and singlet oxygen are approximately 1–3 nm and 3–4 nm, respectively.^5,9)^ Therefore, in the CALI method, the short diffusion of ROS is an important factor in determining the target specificity of the CALI experiment. On the other hand, some photosensitizers, such as riboflavin and flavin mononucleotide, have been reported to be able to induce both Type I and Type II reactions.^10,11)^ Therefore, photosensitizers may not produce only specific ROS. In any case, the target specificity of the CALI experiment needs to be demonstrated individually in various ways.

## 3 Photosensitizers for CALI

### 3-1 Chemical photosensitizers.

Since the development of the CALI method, a number of photosensitizers have been reported. The first photosensitizer used in CALI was malachite green,^4)^ which mainly drives Type I reactions to produce hydroxy radicals in response to red light (620 nm). In the first CALI experiment, the inactivation of biotinylated alkaline phosphatase and β-galactosidase with malachite green-streptavidin was reported *in vitro*. In addition to these purified samples, a malachite green-conjugated antibody against acetylcholinesterase, which was localized on the outside surface of cells, was used for inactivation of acetylcholinesterase on living human erythrocytes. This report, which provided a light manipulation technique for spatiotemporal and causal analysis of molecular function, opened the door to a new era in molecular analysis. Despite these attractive properties, the CALI method with malachite green has not become a common technique because it requires a high-powered pulse laser (*e.g.* average peak power in the original report^4)^ was ∼25 MW/cm^2^), which is not usually available with conventional microscopes. Therefore, for the CALI method to become popular, it was considered necessary to explore a more effective dye for performing CALI with a light source that is commonly installed in the laboratory.

The second generation of CALI dyes was fluorescein.^5,12,13)^ Fluorescein compensates for the weaknesses of malachite green and makes it possible to perform CALI using ordinary microscopic lasers such as continuous wave laser or mercury lamps. By using fluorescein, CALI experiments are also possible in a wider range of fields. For example, fluorescein has been applied to molecular screening of axon tract formation in the lateral olfactory tract (LOT). In the report, the authors established a technique to perform continuous protein inactivation for 24 hours or longer via CALI with fluorescein-labeled monoclonal antibodies against a homogenate of protein extract from the developing LOT and surrounding tissues. By screening these antibodies in organotypic brain slices, LOTUS was identified as an important factor for axonal guidance to antagonize the Nogo receptor.^14)^ On the other hand, although the efficiency of CALI with fluorescein is better than that with malachite green, the ROS production efficiency of fluorescein is still low. Concerns about phototoxicity were also raised because the absorption wavelength (490 nm) was approximately 130 nm shorter than that of malachite green. Therefore, due to the low ROS production efficiency and short excitation wavelengths, there was still room for improvement.

**Figure 3.**
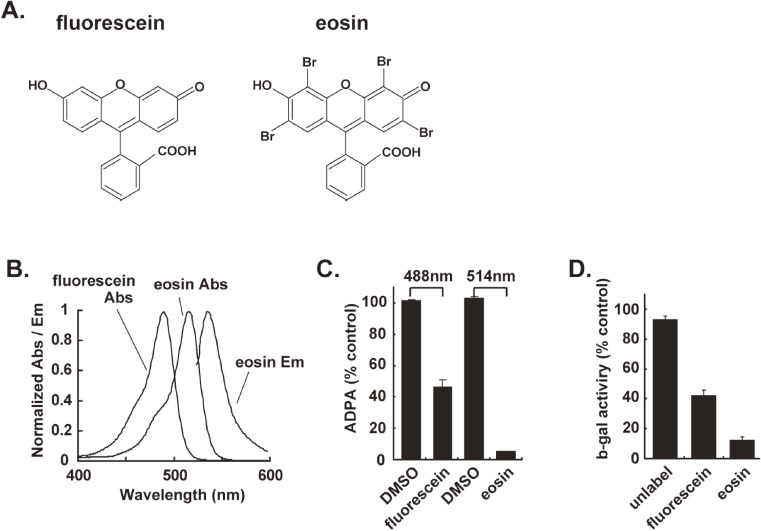
The chemical photosensitizers. **A.** The structure of the fluorescein and eosin. **B.** The spectra of fluorescein and eosin. Abs. and Em. indicate the absorbance spectra and emission spectra, respectively. **C.** Efficiency of singlet oxygen generation *in vitro*. The quenching of ADPA occurs in response to singlet oxygen. **D.** Chromophore-assisted light inactivation (CALI) of the β-galactosidase protein *in vitro* using an anti-β-gal antibody labeled with fluorescein or eosin. This figure was reproduced with modifications based on our original report.^15)^

To obtain the next generation of CALI dyes, our group focused on investigating various compounds with xanthene skeletons (the benzene ring in the center of the anthracene is replaced by a pyran ring) similar to fluorescein. Among many compounds, we found that eosin (2,4,5,7-tetrabromofluorescein, Fig. 3A) has an absorption peak at a wavelength approximately 30 nm (517 nm) longer than that of fluorescein (Fig. 3B), has approximately 11-fold stronger photosensitizing activity (singlet oxygen generation; Fig. 3C), and has an approximately five-fold stronger CALI effect *in vitro* (Fig. 3D).^15)^ As a structural factor, eosin has four Br, which are not present in fluorescein, and these are thought to be responsible for the strong photosensitizing activity.

Recently, NIR (∼666 nm) excitable photosensitizer was developed by improving malachite green with heavy atom substitution to increase intersystem crossing.^16)^ Moreover, the xanthene derivative Janelia Fluor (JF_570_ and JF_549_) has also been reported.^17)^ These dyes are red-shifted compared with malachite green and eosin, and demonstrated sufficient CALI effects by cell ablation of specific neurons *in vivo* or molecular inactivation of PLC-γ. Similar to these, the development of photosensitizers with new characteristics will continue to be important in the future.

### 3-2 Targeting proteins of interest with chemical photosensitizers.

Thus, dyes for CALI have continued to develop in terms of photosensitivity and photosensitizing activity, but how to introduce a photosensitizer to the protein of interest has been a major problem. For example, in the case of cell surface molecules in living cells, antibodies to the extracellular domain of a target molecule can be chemically labeled with a photosensitizer via reaction groups including isothiocyanate groups,^18)^ which react with amines, and maleimide groups,^19)^ which react with thiols. In the case of isothiocyanate, if the pH of the reaction buffer is alkaline, it reacts with the side chain of lysine in an antibody, and if it is neutral, it reacts with the α-amino group. Therefore, it is possible to select a group with higher CALI efficiency by changing the labeling position. However, because antibodies generally do not penetrate cells, applying this method to intracellular molecules requires a technique such as microinjection. To facilitate CALI experiments for molecules inside cells, targeting methods to introduce a chemical photosensitizer to proteins of interest have been reported.

FlAsH-EDT2 and ReAsH-EDT2 are membrane-permeable compounds containing photosensitizers (fluorescein and rhodamine, respectively). Because they react with contiguous tetracysteine sequences, they can target a protein of interest with a photosensitizer simply by fusing the four-cysteine sequence. However, problems such as nonspecific labeling and toxicity have been reported in living cells.^20)^ To improve the targeting specificity in cultured cells, a modified version of them has been reported with improved fluorescence and affinity.^21)^ Moreover, a highly specific system of fluorescein labeling has also been developed using SNAP tags, an enzyme with a molecular weight of 20 kDa that was derived from O^6^-alkylguanine-DNA alkyltransferase.^22)^ Using this technique, CALI of α and γ tubulin was reported in living cells.^23)^ However, in the report, the light intensity during CALI was extremely high (more than 67.5 kJ cm^−2^), and phototoxicity may be a concern for some cells and tissues. These reports called for new technologies that can ensure two things: targeting specificity and low toxicity.

**Figure 4.**
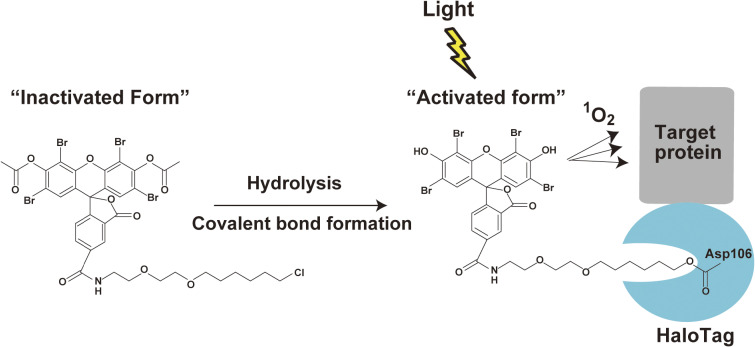
(Color online) Targeting eosin to the protein of interest with the HaloTag labeling technology. The eosin dye for HaloTag, diacetyl-eosin-AM, has two acetyl groups that quench its photoabsorption activity. The diacetyl group is hydrolyzed by cellular esterase after its translocation into the cell by the AM group, which restores its absorption capacity. Note that this technique used the HaloTag7 enzyme to improve the activity and specificity of covalent bond formation. This figure was reproduced with modifications based on our original report.^15)^

Our group reported an eosin-targeting method using HaloTag, an enzyme with a molecular weight of 33 kDa that was developed by improving a dehalogenase, which forms a covalent bond with a compound containing a HaloTag reaction group (HaloTag ligand, Fig. 4).^24)^ HaloTag ligands are cell permeable and used to label target molecules with many fluorescent and nonfluorescent compounds. Thus, by expressing the fusion molecule between the target molecule and HaloTag in cells and adding the HaloTag ligand in the culture medium, the target molecule can be rapidly labeled with the HaloTag ligand. We chemically synthesized a HaloTag ligand with eosin (diacetyl-eosin ligand) and developed an eosin labeling system with high cell permeability and target specificity (Fig. 3B).^15)^ In fact, using this system, we succeeded in optically inactivating PKCγ and AuroraB without any cytotoxicity during CALI. This system has enabled us to establish a simple method for labeling target molecules with eosin and performing CALI. The HaloTag system has also been applied to NIR-excited malachite green derivatives and Janelia Fluor, which were introduced in the previous subsection. A recent study demonstrated the *in vivo* ablation of forebrain neurons using JF_570_ and JF_585_, which were specifically targeted using the HaloTag system. In addition, with a HaloTag system, a “Flexi HaloTag” clone library is available that fuses HaloTag with various genes.^25)^ As these libraries become more complete, it will be possible to use CALI to screen for spatiotemporal functions of proteins during biological events. Examples of molecular inactivation by CALI using chemical photosensitizers are summarized in Table 1.^26–37)^

### 3-3 Genetically encoded photosensitizers.

Chemically synthesized photosensitizers have the advantage of strong activity, and the development of superior methods of targeting photosensitizers such as SNAP-Tag and HaloTag, as described in the previous subsection, has made it possible to apply them to intracellular molecules. However, if the photosensitizer itself is completely encoded by a gene, it is possible to create transgenic or knock-in animals, and the CALI method is expected to become more popular for molecular analysis, especially *in vivo*. In this context, a genetically encoded photosensitizing protein, called KillerRed, has been developed. KillerRed is based on an *Anthomedusae sp.* derived chromoprotein called anm2CP, which was screened for genetic mutations to determine whether it has the ability to kill *E. coli* with light irradiation.^38)^ In the original report, KillerRed was able to generate both superoxide radicals and singlet oxygen, as revealed by specific ROS indicators and quenchers,^38)^ and similar results were confirmed in other reports.^39)^ In contrast, another study was unable to detect singlet oxygen generation^40)^ or to detect the effect of Trolox, a singlet oxygen quencher,^41)^ and the effect of D_2_O, which is known to increase the lifetime of singlet oxygen.^42)^ Therefore, it is assumed that the production of singlet oxygen by KillerRed is opaque, and it may depend on the specificity of the detection system. It is also possible that singlet oxygen generation by KillerRed may depend on the experimental conditions, such as the intensity of the irradiated light. Regardless of the type of ROS produced, the key to CALI experiments is target specificity.

For CALI with KillerRed, all that is necessary is to express a fusion molecule with a protein of interest in a specific cell using an expression vector and then irradiate it with light. Although it is not possible to perform CALI of endogenous molecules via this method alone, it is a groundbreaking method that enables extremely simple CALI experiments. On the other hand, KillerRed is the world’s first genetically encoded photosensitizer, but the problem of nonphysiological localization of the fused target molecule was observed because KillerRed is a dimeric molecule. For example, when the target molecule was fused with KillerRed, nonphysiological localization (fibrillarin), aggregation (keratin and Cx43), and cytotoxicity (H2B) were also observed.^39)^ Although tandem-KillerRed was devised to solve these problems, monomerization of KillerRed was strongly desired because it is quite large (∼53 kDa) for a tag.

In response to this, our group has successfully developed a monomeric KillerRed, SuperNova, by means of structure prediction and random mutation screening, which improved on the weaknesses of KillerRed, such as nonphysiological localization and toxicity.^39)^ SuperNova also enabled CALI experiments for cofilin, a regulatory protein for actin dynamics. Moreover, the chromophore of KillerRed and SuperNova are also completely encoded by the gene and its structure is similar to that of GFP. Therefore, it may be possible to create color variants. For example, a mutant Killer-Orange with maximum absorption at 455 nm was reported.^43)^ In addition, a mutant SuperNova-Green with maximum absorption at 440 nm was also developed by introducing a mutation containing Y66W and V44A in and around its chromophore.^44)^ This is expected to make it possible to multitask the CALI method, where multiple molecules can be manipulated with different wavelengths in the living cell. Because SuperNova has many advantages compared with KillerRed, as described above, it is gradually being applied for *in vitro* and *in vivo* CALI experiments (Tables 2 and 3).

In addition to KillerRed and SuperNova, a monomeric photosensitizing protein, miniSOG, has been developed based on the LOV domain of Phototoropinv2.^40)^ Unlike KillerRed and SuperNova, miniSOG is genetically encoded except for its chromophore, flavin mononucleotide (FMN). Because miniSOG is a small protein only consisting of 106 amino acids, it is unlikely that folding and structural inhibition of protein interactions will occur. Another advantage of miniSOG is that it exhibits high efficiency singlet oxygen generation (Φ = 0.47) compared with previously reported photosensitizing proteins. Although miniSOG has great advantages as described above, it is likely to be difficult to produce color variants because the chromophore is an FMN. Moreover, although FMNs are considered to be abundant in most cells, it is important to confirm whether FMNs are actually contained in the tissue when conducting CALI to avoid misinterpretation of the experimental results.^45)^ In addition to its application in CALI, miniSOG has also been used for detection using osmium tetroxide via electron microscopy.^46)^ For example, by fusing miniSOG to a protein of interest and expressing it in the cell, the distribution of the target molecule can be easily analyzed by electron microscopy without the use of antibodies.

As mentioned above, various photosensitizing proteins have been reported so far. To date, many of the molecules have been inactivated by CALI using photosensitizing proteins. The main examples are summarized in Table 2.^47–60)^ Although the CALI method has been mainly used *in vitro*, such as in cultured cells, recently, there have been an increasing number of reports using the CALI method *in vivo*.

## 4 Applications of CALI to inactivate specific molecules *in vivo*

As CALI technology has matured, a number of studies have applied CALI *in vivo* to perform spatiotemporal analysis of molecular functions in model animals. Here, some recent examples will be introduced of the *in vivo* photoinactivation of specific molecules, which will provide a better understanding of the characteristics and advantages of the CALI method.

Because *C. elegans* is a transparent animal, it is a good animal model for optical measurements, such as live imaging and optical manipulation. By fusing miniSOG to UNC-13, a regulatory protein for synaptic vesicle release in the active zone, it was revealed that UNC-13 is involved in the spontaneous release and the fast phase of evoked release via acute and local inactivation by CALI in live animals.^61)^ In addition, CALI of VAMP2 and synaptophysin, including the SNARE complex, was also reported to reduce synaptic release and behavioral changes.^62)^ In *C. elegans*, CALI experiments by SuperNova in the macromolecules, a mitochondrial electron transport chain complex II protein, SDHB and SDHC with CRISPR-Cas9 knock-in technique have been reported.^63)^ In this report, the specificity of CALI was investigated by measuring the activities of neighboring proteins, such as complex I (NADPH), complex IV (cytochrome c oxidase), and citrate synthase. As described in the first section, such control experiments to test the specificity of CALI is important for showing the reliability of the study.

After several reports in *C. elegans*, CALI technology has entered the field of developmental biology in *Drosophila*. In genitalia rotation of *Drosophila* during development, epithelial cell-cell junction remodeling involves the shortening and loss of bicellular junctions and the subsequent growth of bicellular junctions in a new direction. Using SuperNova gene knock-in at the C-terminal of the myosin II, spatial-temporal inactivation of myosin II was induced at or around remodeling junctions.^64)^ This study clearly showed the physiological role of myosin II in the growth of cell junctions during genitalia rotation. This study also demonstrated the efficacy of the CALI technique with SuperNova for endogenous proteins.

**Figure 5.**
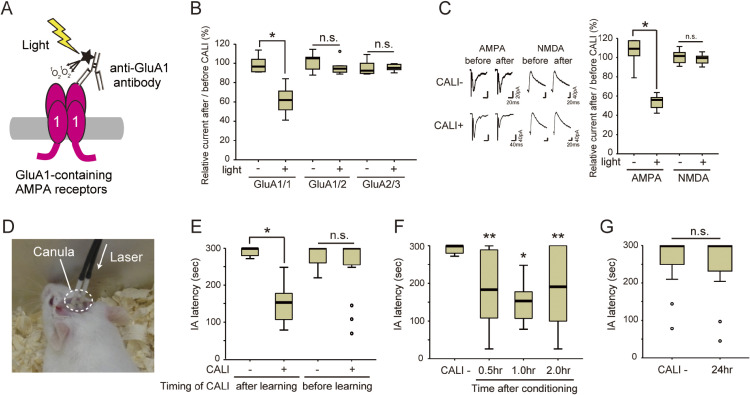
(Color online) *In vivo* chromophore-assisted light inactivation (CALI) for GluA1 homomeric AMPA receptors. **A.** Schematic representation of this technology. **B.** CALI for 3 types of AMPA receptor complexes in CHO cells. *p < 0.001; unpaired two-tailed *t*-test. n.s. indicates no significance. **C.** CALI for synaptic AMPA receptors. Note that the NMDA receptor is a neighboring protein in synapses that was used as a negative control to show the target specificity of CALI. *p < 0.001; unpaired two-tailed *t*-test. n.s. indicates no significance. **D.** Procedure for *in vivo* CALI. **E.** *In vivo* CALI before and after learning. *p < 0.001 in after learning (Mann–Whitney *U* test). n.s. indicates no significance. **F**, **G.** Time course analysis (0–24 hrs after learning) of *in vivo* CALI. Note that the CALI effect was limited in the early phase of learning (0.5–2 hrs after learning). This figure was reproduced with modifications based on our original report.^69)^ *p < 0.01 vs. CALI−, **p < 0.05 vs. CALI− (Kruskal–Wallis test by *post hoc* analysis with Dunne’s test). n.s. indicates no significance.

Our group presented a new possibility of using CALI technology for neuroscience experiments in mammals. The AMPA receptor is important for the synaptic response in excitatory synapses. In the adult hippocampus, this receptor is expressed as 3 subunits, GluA1, GluA2, and GluA3, which combine to form a complex of GluA1/1, GluA1/2, and GluA2/3.^65)^ Among these complexes, GluA1-containing receptors are delivered into synapses in response to learning and experience *in vivo*.^66–68)^ However, the physiological role of each complex remains unknown *in vivo*, especially in hippocampal-dependent memory formation. To address this issue, we developed a CALI method for the endogenous AMPA receptor GluA1 using a specific antibody against its extracellular domain (Fig. 5A).^69)^ After screening monoclonal antibodies, we developed a CALI method that was able to specifically inactivate the GluA1 homomeric receptor, GluA1/1 (Fig. 5B). Because the efficiency of CALI should be due to the steric structure, as described in the first section, this specificity may be due to structural differences between GluA1/1 and other complexes. Moreover, the target specificity of CALI in synapses was confirmed using control experiments with NMDA receptors (Fig. 5C). By introducing this technology *in vivo*, we found that CALI of GluA1/1 eliminated fear memory in the early stages, 0.5–2 hours after learning, suggesting that GluA1/1 is important for the acquisition of hippocampal fear memory (Fig. 5D–G).^69)^ Based on these results, this technology will be suitable for analyzing the early phase of learning, such as the formation of a memory engram. Because this CALI technology was also applied in hypothalamus-habenula synapses to reveal the mechanism of associative learning *in vivo*,^70)^ further applications in various brain regions are expected in the future. This technology is now widely regarded as the next generation of synaptic analysis in living animals.^71–73)^ Examples of molecular inactivation by CALI *in vivo* are also summarized in Table 3.^61,74–78)^

## 5 Future innovations in CALI

As mentioned above, the CALI method is gradually becoming a well-known molecular analysis method through various technical improvements. One important point for the development of future CALI methods will be the ability to manipulate multiple molecules simultaneously. To date, only one-color variants have been reported for SuperNova and KillerRed.^43,44)^ In the future, it is desired to establish a multicolored technology, similar to GFP, which can manipulate the behavior of many molecules simultaneously.

In a recent study, a high-throughput imaging technique for molecules in the nervous system was reported by combining CRISPR-Cas9 and GFP.^79,80)^ This is a remarkable new technology that in principle allows genome-wide molecular visualization. For example, can we apply the CALI method to various molecules by combining photosensitizing proteins in a similar way? It might seem difficult to make this possible. As mentioned before, for the photosensitizer to be effective, amino acid residues that break the structure of the target molecule upon oxidation should be inside the diffusion radius of the ROS. That is, the relative position of the photosensitizer to the target molecule is important for CALI efficiency. In support of this idea, we have found in previous studies that even for monoclonal antibodies (labeled by eosin) with the same epitope, the efficiency of CALI varies for each antibody, probably because the relative position between the photosensitizer and target protein should be different for each antibody.^69)^ Thus, unlike in the case of using GFP for live imaging, the effect of CALI with a photosensitizing protein can be presumed to be less certain only when fused to the target molecule. Therefore, future development of innovative CALI technologies that allow for genome-wide and high-throughput optical manipulation of various molecules will be strongly desired.

In addition to such basic research, the CALI method is also expected to have medical applications such as photodynamic therapy (PDT).^81)^ The photosensitizers used in PDT, such as porphyrin compounds, have the ability to accumulate in cancer cells, and the efficiency of PDT depends on the efficiency of ROS generation as well as the ability to perform PDT in deep tissue. The current photosensitizers used in PDT are porphyrins, which have a photosensitive wavelength of approximately 690 nm. Furthermore, a photo-immunotherapy using new photosensitizer, IR700 (excited at 690 nm), has been reported as a principle similar to the CALI method.^82,83)^ Because these excitation wavelengths are still short, these are currently limited to the tissue surface, such as skin cancer. Therefore, the development of new photosensitizers that can be excited at longer wavelengths is needed in the future. This is an important point in basic research as well. For example, when irradiating deep into the brain, such as in the basal ganglia, it is necessary to insert the optical cannula deep into the brain, which is a relatively invasive experiment. Therefore, if a photosensitizer with long wavelengths can be developed, it is expected that CALI can be performed deep in the brain without a cannula insertion in the future, making less invasive experiments possible.

The CALI method is not as popular as other light manipulation methods, such as channel rhodopsin, which has become indispensable in neuroscience. On the other hand, the CALI method has the great advantage of being able to specifically inactivate molecules, so it is expected to become more widespread in various research fields due to technical improvements in the near future.

## Figures and Tables

**Table 1.** Examples of molecular inactivation by CALI with a chemical photosensitizer in vitro

Photosensitizer	Protein of interest	Function	Targeting method	CALI sample	Reference
Malachite Green	Acetylcholinesterase	Hydrolysis of acetylcholine	Antibody	*In vitro*	4
	Calcineurin	Neurite outgrowth	Antibody	*In vitro*	26
	IP3R	Calcium signal	Antibody	*In vitro*	27
	RNA	Nucleotide	RNA aptamer	*In vitro*	28
	L1	Neurite outgrowth	Antibody	*In vitro*	29
	NCAM-180	Growth cone protrusion	Antibody	*In vitro*	29
	pp60c-src	Inhibition of neurite outgrowth	Antibody	*In vitro*	30
	Myosin II	Neurite outgrowth	Antibody	*In vitro*	31
	IP3R	Calcium signal	Ligand conjugation	*In vitro*	32
	CRMP1/2	Neurite outgrowth	Antibody	*In vitro*	33

Fluorescein	β1 integrin	Cell invasion	Antibody	*In vitro*	5
	Ki-67	rRNA synthesis	Antibody	*In vitro*	34
	α-/γ-Tubulin	Spindle formation	SNAP-Tag	*In vitro*	22
	LOTUS	Nogo-R antagonist	Antibody	Brain slice	14
	5-HT3A	Serotonin receptor	Binding peptide	*In vitro*	35
	Kinesin	Motor protein	Antibody	*In vitro*	13
	V-ATPase V0c	Neurotransmitter release	FlAsH	Brain slice	36

Eosin	PKC-γ	Serine threonine kinase	HaloTag	*In vitro*	15
	AuroraB	Cell division	HaloTag	*In vitro*	15
	RBBP9	Serine hydrogenase	Binding peptide	*In vitro*	37

**Table 2.** Examples of molecular inactivation by CALI using photosensitizing proteins in vitro

Photosensitizer	Protein of interest	Function	Targeting method	CALI sample	Reference
KillerRed	PLCδ1	Lipid metabolism *etc.*	Fusion	*In vitro*	38
	β1-integrin	Invadasome structure	Fusion	*In vitro*	47
	Centrin2	Replication of centromere	Fusion	Brain slice	48
	Histon H2B	Component of nucleosome	Fusion	*In vitro*	49

	RBMX	Chromosome morphogenesis	Fusion	*In vitro*	50
	Sec13	Biogenesis of COPII-coated vesicle	Fusion	*In vitro*	51
	Aquaporin1/4	Water transport	Fusion	*In vitro*	52
	Cofirin	Actin filament disassembly	Fusion	*In vitro*	53
	Rab7	Endocytosis	Fusion	*In vitro*	54
	GRASP55/65	Formation of Golgi ribbon	Fusion	*In vitro*	55
Tandem-KillerRed	Ran	Membrane targeting of RhoA	Fusion	*In vitro*	56

SuperNova	Cofirin	Actin filament disassembly	Fusion	*In vitro*	15
	mDia1	Rho effector *etc.*	Fusion	*In vitro*	57
	CamKIIβ	LTP induction	Fusion	Brain slice	58
	Synapsin	Component of synaptic vesicle	Fusion	*In vitro*	59
	Synaptophysin	Component of synaptic vesicle	Fusion	*In vitro*	59
	Arl13b	Primary cilium formation	Fusion	Chick embryonic slice	60
SuperNova-Green	PLCδ1	Lipid metabolism *etc.*	Fusion	*In vitro*	44

miniSOG	VAMP2	SNARE protein	Fusion	Brain slice	62
	SYP1	SNARE protein	Fusion	Brain slice	62

**Table 3.** Examples of molecular inactivation by CALI using photosensitizing proteins in vivo

Photosensitizer	Protein of interest	Function	Targeting method	CALI sample	Reference
Malachite Green	Patched	Neuronal cell fate	Antibody	*Drosophila*	74

Fluorescein	Synaptotagmin I	Neurotransmitter release	FlAsH	*Drosophila*	75

Eosin	AMPA-R GluA1/1	Excitatory synaptic response	Antibody	*Mouse* (hippocampus)	69
	AMPA-R GluA1/1	Excitatory synaptic response	Antibody	*Mouse* (lateral habenula)	70

KillerRed	GON domain	Protein secretion from ER	Fusion	*C. elegans*	76

SuperNova	SDHB/SDHC	Mitochondrial electron transport chain complex II	Fusion(CRISPR)	*C. elegans*	63
	Myosin II	Growth of cell junction	Fusion(CRISPR)	*Drosophila*	64

miniSOG	VAMP2	SNARE protein	Fusion	*C. elegans*	62
	UNC-13	Synaptic vesicle release	Fusion	*C. elegans*	61
	PTRN-1	Axon regeneration	Fusion	*C. elegans*	77
	MEV-1	Mitochondrial electron transport chain complex II	Fusion	*C. elegans*	78

## Nonstandard abbreviation list

CALI: chromophore-assisted light inactivation
FMN: flavin mononucleotide
GFP: green fluorescent protein
HO^•^: hydroxyl radical
LOT: lateral olfactory tract
^1^O_2_: singlet oxygen
O_2_^•−^: superoxide radical
PDT: photodynamic therapy
PS*: T1 photosensitizer
ROS: reactive oxygen species
T_1_: triplet state

## References

[1] Murakoshi H. Wang H. Yasuda R. (2011) Local, persistent activation of Rho GTPases during plasticity of single dendritic spines. Nature 472, 100–104. 21423166 10.1038/nature09823 PMC3105377

[2] Takemoto K. Kuranaga E. Tonoki A. Nagai T. Miyawaki A. Miura M. (2007) Local initiation of caspase activation in Drosophila salivary gland programmed cell death in vivo. Proc. Natl. Acad. Sci. U.S.A. 104, 13367–13372. 17679695 10.1073/pnas.0702733104 PMC1948907

[3] Oda Y. Fukuda H. (2012) Initiation of cell wall pattern by a Rho- and microtubule-driven symmetry breaking. Science 337, 1333–1336. 22984069 10.1126/science.1222597

[4] Jay D.G. (1988) Selective destruction of protein function by chromophore-assisted laser inactivation. Proc. Natl. Acad. Sci. U.S.A. 85, 5454–5458. 3399501 10.1073/pnas.85.15.5454 PMC281775

[5] Beck S. Sakurai T. Eustace B.K. Beste G. Schier R. Rudert F. (2002) Fluorophore-assisted light inactivation: A high-throughput tool for direct target validation of proteins. Proteomics 2, 247–255. 11921440 10.1002/1615-9861(200203)2:3<247::aid-prot247>3.0.co;2-k

[6] Quinn J.C. Kessell A. Weston L.A. (2014) Secondary plant products causing photosensitization in grazing herbivores: Their structure, activity and regulation. Int. J. Mol. Sci. 15, 1441–1465. 24451131 10.3390/ijms15011441 PMC3907879

[7] Wojtovich A.P. Foster T.H. (2014) Optogenetic control of ROS production. Redox Biol. 2, 368–376. 24563855 10.1016/j.redox.2014.01.019 PMC3926119

[8] Kuss-Petermann M. Wenger O.S. (2016) Electron transfer rate maxima at large donor–acceptor distances. J. Am. Chem. Soc. 138, 1349–1358. 26800279 10.1021/jacs.5b11953

[9] Liao J.C. Roider J. Jay D.G. (1994) Chromophore-assisted laser inactivation of proteins is mediated by the photogeneration of free radicals. Proc. Natl. Acad. Sci. U.S.A. 91, 2659–2663. 8146171 10.1073/pnas.91.7.2659 PMC43429

[10] Lee P.C.C. Rodgers M.A.J. (1987) Laser flash photokinetic studies of rose bengal sensitized photodynamic interactions of nucleotides and DNA. Photochem. Photobiol. 45, 79–86. 3031708 10.1111/j.1751-1097.1987.tb08407.x

[11] Barnett M.E. Baran T.M. Foster T.H. Wojtovich A.P. (2018) Quantification of light-induced miniSOG superoxide production using the selective marker, 2-hydroxyethidium. Free Radic. Biol. Med. 116, 134–140. 29353158 10.1016/j.freeradbiomed.2018.01.014 PMC5815924

[12] Jean B. Schmolz M.W. Schollhorn V.G. (1992) Selective laser-induced inactivation of proteins (SLIP) by labelling with chromophores. Med. Biol. Eng. Comput. 30, CE17–CE20. 1487929 10.1007/BF02446173

[13] Surrey T. Elowitz M.B. Wolf P.E. Yang F. Nedelec F. Shokat K. (1998) Chromophore-assisted light inactivation and self-organization of microtubules and motors. Proc. Natl. Acad. Sci. U.S.A. 95, 4293–4298. 9539730 10.1073/pnas.95.8.4293 PMC22482

[14] Sato Y. Iketani M. Kurihara Y. Yamaguchi M. Yamashita N. Nakamura F. (2011) Cartilage acidic protein-1B (LOTUS), an endogenous Nogo receptor antagonist for axon tract formation. Science 333, 769–773. 21817055 10.1126/science.1204144 PMC3244695

[15] Takemoto K. Matsuda T. McDougall M. Klaubert D.H. Hasegawa A. Los G.V. (2011) Chromophore-assisted light inactivation of HaloTag fusion proteins labeled with eosin in living cells. ACS Chem. Biol. 6, 401–406. 21226520 10.1021/cb100431e

[16] He J. Wang Y. Missinato M.A. Onuoha E. Perkins L.A. Watkins S.C. (2016) A genetically targetable near-infrared photosensitizer. Nat. Method. 13, 263–268. 10.1038/nmeth.3735 PMC4916159 26808669

[17] Binns T.C. Ayala A.X. Grimm J.B. Tkachuk A.N. Castillon G.A. Phan S. (2020) Rational design of bioavailable photosensitizers for manipulation and imaging of biological systems. Cell Chem. Biol. 27, 1063–1072. e1067. 32698018 10.1016/j.chembiol.2020.07.001 PMC7483975

[18] Rinderknecht H. (1962) Ultra-rapid fluorescent labelling of proteins. Nature 193, 167–168. 10.1038/193167b0 14492332

[19] Renault K. Fredy J.W. Renard P.Y. Sabot C. (2018) Covalent modification of biomolecules through maleimide-based labeling strategies. Bioconjug. Chem. 29, 2497–2513. 29954169 10.1021/acs.bioconjchem.8b00252

[20] Stroffekova K. Proenza C. Beam K.G. (2001) The protein-labeling reagent FLASH-EDT2 binds not only to CCXXCC motifs but also non-specifically to endogenous cysteine-rich proteins. Pflugers Arch. 442, 859–866. 11680618 10.1007/s004240100619

[21] Martin B.R. Giepmans B.N. Adams S.R. Tsien R.Y. (2005) Mammalian cell-based optimization of the biarsenical-binding tetracysteine motif for improved fluorescence and affinity. Nat. Biotechnol. 23, 1308–1314. 16155565 10.1038/nbt1136

[22] Keppler A. Pick H. Arrivoli C. Vogel H. Johnsson K. (2004) Labeling of fusion proteins with synthetic fluorophores in live cells. Proc. Natl. Acad. Sci. U.S.A. 101, 9955–9959. 15226507 10.1073/pnas.0401923101 PMC454197

[23] Keppler A. Ellenberg J. (2009) Chromophore-assisted laser inactivation of α- and γ-tubulin SNAP-tag fusion proteins inside living cells. ACS Chem. Biol. 4, 127–138. 19191588 10.1021/cb800298u

[24] Los G.V. Wood K. (2007) The HaloTag: A novel technology for cell imaging and protein analysis. Methods Mol. Biol. 356, 195–208. 16988404 10.1385/1-59745-217-3:195

[25] Nagase T. Yamakawa H. Tadokoro S. Nakajima D. Inoue S. Yamaguchi K. (2008) Exploration of human ORFeome: High-throughput preparation of ORF clones and efficient characterization of their protein products. DNA Res. 15, 137–149. 18316326 10.1093/dnares/dsn004 PMC2650635

[26] Chang H.Y. Takei K. Sydor A.M. Born T. Rusnak F. Jay D.G. (1995) Asymmetric retraction of growth cone filopodia following focal inactivation of calcineurin. Nature 376, 686–690. 7544441 10.1038/376686a0

[27] Takei K. Shin R.M. Inoue T. Kato K. Mikoshiba K. (1998) Regulation of nerve growth mediated by inositol 1,4,5-trisphosphate receptors in growth cones. Science 282, 1705–1708. 9831561 10.1126/science.282.5394.1705

[28] Grate D. Wilson C. (1999) Laser-mediated, site-specific inactivation of RNA transcripts. Proc. Natl. Acad. Sci. U.S.A. 96, 6131–6136. 10339553 10.1073/pnas.96.11.6131 PMC26847

[29] Takei K. Chan T.A. Wang F.S. Deng H. Rutishauser U. Jay D.G. (1999) The neural cell adhesion molecules L1 and NCAM-180 act in different steps of neurite outgrowth. J. Neurosci. 19, 9469–9479. 10531450 10.1523/JNEUROSCI.19-21-09469.1999 PMC6782940

[30] Hoffman-Kim D. Kerner J.A. Chen A. Xu A. Wang T.-F. Jay D.G. (2002) pp60c-src is a negative regulator of laminin-1-mediated neurite outgrowth in chick sensory neurons. Mol. Cell. Neurosci. 21, 81–93. 12359153 10.1006/mcne.2002.1157

[31] Diefenbach T.J. Latham V.M. Yimlamai D. Liu C.A. Herman I.M. Jay D.G. (2002) Myosin 1c and myosin IIB serve opposing roles in lamellipodial dynamics of the neuronal growth cone. J. Cell Biol. 158, 1207–1217. 12356865 10.1083/jcb.200202028 PMC2173244

[32] Inoue T. Kikuchi K. Hirose K. Iino M. Nagano T. (2003) Spatiotemporal laser inactivation of inositol 1,4,5-trisphosphate receptors using synthetic small-molecule probes. Chem. Biol. 10, 503–509. 12837383 10.1016/s1074-5521(03)00122-4

[33] Higurashi M. Iketani M. Takei K. Yamashita N. Aoki R. Kawahara N. (2012) Localized role of CRMP1 and CRMP2 in neurite outgrowth and growth cone steering. Dev. Neurobiol. 72, 1528–1540. 22378692 10.1002/dneu.22017

[34] Rahmanzadeh R. Hüttmann G. Gerdes J. Scholzen T. (2007) Chromophore-assisted light inactivation of pKi-67 leads to inhibition of ribosomal RNA synthesis. Cell Prolif. 40, 422–430. 17531085 10.1111/j.1365-2184.2007.00433.x PMC6496591

[35] Morton R.A. Luo G. Davis M.I. Hales T.G. Lovinger D.M. (2011) Fluorophore assisted light inactivation (FALI) of recombinant 5-HT3A receptor constitutive internalization and function. Mol. Cell. Neurosci. 47, 79–92. 21338684 10.1016/j.mcn.2011.02.007 PMC3172681

[36] Rama S. Boumedine-Guignon N. Sangiardi M. Youssouf F. Maulet Y. Lévêque C. (2019) Chromophore-assisted light inactivation of the V-ATPase V0c subunit inhibits neurotransmitter release downstream of synaptic vesicle acidification. Mol. Neurobiol. 56, 3591–3602. 30155790 10.1007/s12035-018-1324-1

[37] Liu X. Dix M. Speers A.E. Bachovchin D.A. Zuhl A.M. Cravatt B.F. (2012) Rapid development of a potent photo-triggered inhibitor of the serine hydrolase RBBP9. ChemBioChem 13, 2082–2093. 22907802 10.1002/cbic.201200445 PMC3777423

[38] Bulina M.E. Chudakov D.M. Britanova O.V. Yanushevich Y.G. Staroverov D.B. Chepurnykh T.V. (2006) A genetically encoded photosensitizer. Nat. Biotechnol. 24, 95–99. 16369538 10.1038/nbt1175

[39] Takemoto K. Matsuda T. Sakai N. Fu D. Noda M. Uchiyama S. (2013) SuperNova, a monomeric photosensitizing fluorescent protein for chromophore-assisted light inactivation. Sci. Rep. 3, 2629. 24043132 10.1038/srep02629 PMC3775092

[40] Shu X. Lev-Ram V. Deerinck T.J. Qi Y. Ramko E.B. Davidson M.W. (2011) A genetically encoded tag for correlated light and electron microscopy of intact cells, tissues, and organisms. PLoS Biol. 9, e1001041. 21483721 10.1371/journal.pbio.1001041 PMC3071375

[41] Wang B. Van Veldhoven P.P. Brees C. Rubio N. Nordgren M. Apanasets O. (2013) Mitochondria are targets for peroxisome-derived oxidative stress in cultured mammalian cells. Free Radic. Biol. Med. 65, 882–894. 23988789 10.1016/j.freeradbiomed.2013.08.173

[42] Serebrovskaya E.O. Edelweiss E.F. Stremovskiy O.A. Lukyanov K.A. Chudakov D.M. Deyev S.M. (2009) Targeting cancer cells by using an antireceptor antibody-photosensitizer fusion protein. Proc. Natl. Acad. Sci. U.S.A. 106, 9221–9225. 19458251 10.1073/pnas.0904140106 PMC2695119

[43] Sarkisyan K.S. Zlobovskaya O.A. Gorbachev D.A. Bozhanova N.G. Sharonov G.V. Staroverov D.B. (2015) KillerOrange, a genetically encoded photosensitizer activated by blue and green light. PLoS One 10, e0145287. 26679300 10.1371/journal.pone.0145287 PMC4683004

[44] Riani Y.D. Matsuda T. Takemoto K. Nagai T. (2018) Green monomeric photosensitizing fluorescent protein for photo-inducible protein inactivation and cell ablation. BMC Biol. 16, 50. 29712573 10.1186/s12915-018-0514-7 PMC5928576

[45] Ryumina A.P. Serebrovskaya E.O. Shirmanova M.V. Snopova L.B. Kuznetsova M.M. Turchin I.V. (2013) Flavoprotein miniSOG as a genetically encoded photosensitizer for cancer cells. Biochim. Biophys. Acta 1830, 5059–5067. 23876295 10.1016/j.bbagen.2013.07.015

[46] Atasoy D. Betley J.N. Li W.P. Su H.H. Sertel S.M. Scheffer L.K. (2014) A genetically specified connectomics approach applied to long-range feeding regulatory circuits. Nat. Neurosci. 17, 1830–1839. 25362474 10.1038/nn.3854 PMC4292906

[47] Destaing O. Planus E. Bouvard D. Oddou C. Badowski C. Bossy V. (2010) β1A integrin is a master regulator of invadosome organization and function. Mol. Biol. Cell 21, 4108–4119. 20926684 10.1091/mbc.E10-07-0580 PMC2993740

[48] de Anda F.C. Meletis K. Ge X. Rei D. Tsai L.-H. (2010) Centrosome motility is essential for initial axon formation in the neocortex. J. Neurosci. 30, 10391. 20685982 10.1523/JNEUROSCI.0381-10.2010 PMC6634663

[49] Serebrovskaya E.O. Gorodnicheva T.V. Ermakova G.V. Solovieva E.A. Sharonov G.V. Zagaynova E.V. (2011) Light-induced blockage of cell division with a chromatin-targeted phototoxic fluorescent protein. Biochem. J. 435, 65–71. 21214518 10.1042/BJ20101217

[50] Matsunaga S. Takata H. Morimoto A. Hayashihara K. Higashi T. Akatsuchi K. (2012) RBMX: A regulator for maintenance and centromeric protection of sister chromatid cohesion. Cell Rep. 1, 299–308. 22832223 10.1016/j.celrep.2012.02.005

[51] Jarvela T. Linstedt A.D. (2012) Irradiation-induced protein inactivation reveals Golgi enzyme cycling to cell periphery. J. Cell. Sci. 125, 973–980. 22421362 10.1242/jcs.094441 PMC3311931

[52] Baumgart F. Rossi A. Verkman A.S. (2012) Light inactivation of water transport and protein-protein interactions of aquaporin-Killer Red chimeras. J. Gen. Physiol. 139, 83–91. 22200949 10.1085/jgp.201110712 PMC3250104

[53] Vitriol E.A. Wise A.L. Berginski M.E. Bamburg J.R. Zheng J.Q. (2013) Instantaneous inactivation of cofilin reveals its function of F-actin disassembly in lamellipodia. Mol. Biol. Cell 24, 2238–2247. 23676663 10.1091/mbc.E13-03-0156 PMC3708729

[54] Serebrovskaya E.O. Ryumina A.P. Boulina M.E. Shirmanova M.V. Zagaynova E.V. Bogdanova E.A. (2014) Phototoxic effects of lysosome-associated genetically encoded photosensitizer KillerRed. J. Biomed. Opt. 19, 071403. 24365992 10.1117/1.JBO.19.7.071403

[55] Jarvela T. Linstedt A.D. (2014) Isoform-specific tethering links the Golgi ribbon to maintain compartmentalization. Mol. Biol. Cell 25, 133–144. 24227884 10.1091/mbc.E13-07-0395 PMC3873884

[56] Zaoui K. Boudhraa Z. Khalifé P. Carmona E. Provencher D. Mes-Masson A.-M. (2019) Ran promotes membrane targeting and stabilization of RhoA to orchestrate ovarian cancer cell invasion. Nat. Commun. 10, 2666. 31209254 10.1038/s41467-019-10570-w PMC6573066

[57] Isogai T. van der Kammen R. Leyton-Puig D. Kedziora K.M. Jalink K. Innocenti M. (2015) Initiation of lamellipodia and ruffles involves cooperation between mDia1 and the Arp2/3 complex. J. Cell Sci. 128, 3796–3810. 26349808 10.1242/jcs.176768

[58] Kim K. Lakhanpal G. Lu H.E. Khan M. Suzuki A. Hayashi M.K. (2015) A temporary gating of actin remodeling during synaptic plasticity consists of the interplay between the kinase and structural functions of CaMKII. Neuron 87, 813–826. 26291163 10.1016/j.neuron.2015.07.023 PMC4548268

[59] Hoffmann S. Orlando M. Andrzejak E. Bruns C. Trimbuch T. Rosenmund C. (2019) Light-activated ROS production induces synaptic autophagy. J. Neurosci. 39, 2163. 30655355 10.1523/JNEUROSCI.1317-18.2019 PMC6433757

[60] Toro-Tapia G. Das R.M. (2020) Primary cilium remodeling mediates a cell signaling switch in differentiating neurons. Sci. Adv. 6, eabb0601. 32494754 10.1126/sciadv.abb0601 PMC7252506

[61] Zhou K. Stawicki T.M. Goncharov A. Jin Y. (2013) Position of UNC-13 in the active zone regulates synaptic vesicle release probability and release kinetics. eLife 2, e01180. 24220508 10.7554/eLife.01180 PMC3821175

[62] Lin J.Y. Sann S.B. Zhou K. Nabavi S. Proulx C.D. Malinow R. (2013) Optogenetic inhibition of synaptic release with chromophore-assisted light inactivation (CALI). Neuron 79, 241–253. 23889931 10.1016/j.neuron.2013.05.022 PMC3804158

[63] Trewin A.J. Bahr L.L. Almast A. Berry B.J. Wei A.Y. Foster T.H. (2019) Mitochondrial reactive oxygen species generated at the complex-II matrix or intermembrane space microdomain have distinct effects on redox signaling and stress sensitivity in *Caenorhabditis elegans*. Antioxid. Redox Signal. 31, 594–607. 30887829 10.1089/ars.2018.7681 PMC6657295

[64] Uechi H. Kuranaga E. (2019) The tricellular junction protein sidekick regulates vertex dynamics to promote bicellular junction extension. Dev. Cell 50, 327–338. e325. 31353316 10.1016/j.devcel.2019.06.017

[65] Kessels H.W. Malinow R. (2009) Synaptic AMPA receptor plasticity and behavior. Neuron 61, 340–350. 19217372 10.1016/j.neuron.2009.01.015 PMC3917551

[66] Takahashi T. Svoboda K. Malinow R. (2003) Experience strengthening transmission by driving AMPA receptors into synapses. Science 299, 1585–1588. 12624270 10.1126/science.1079886

[67] Mitsushima D. Ishihara K. Sano A. Kessels H.W. Takahashi T. (2011) Contextual learning requires synaptic AMPA receptor delivery in the hippocampus. Proc. Natl. Acad. Sci. U.S.A. 108, 12503–12508. 21746893 10.1073/pnas.1104558108 PMC3145714

[68] Rumpel S. LeDoux J. Zador A. Malinow R. (2005) Postsynaptic receptor trafficking underlying a form of associative learning. Science 308, 83–88. 15746389 10.1126/science.1103944

[69] Takemoto K. Iwanari H. Tada H. Suyama K. Sano A. Nagai T. (2017) Optical inactivation of synaptic AMPA receptors erases fear memory. Nat. Biotechnol. 35, 38–47. 27918547 10.1038/nbt.3710

[70] Trusel M. Nuno-Perez A. Lecca S. Harada H. Lalive A.L. Congiu M. (2019) Punishment-predictive cues guide avoidance through potentiation of hypothalamus-to-habenula synapses. Neuron 102, 120–127. e124. 30765165 10.1016/j.neuron.2019.01.025

[71] Humeau Y. Choquet D. (2019) The next generation of approaches to investigate the link between synaptic plasticity and learning. Nat. Neurosci. 22, 1536–1543. 31477899 10.1038/s41593-019-0480-6

[72] Frank J.A. Antonini M.-J. Anikeeva P. (2019) Next-generation interfaces for studying neural function. Nat. Biotechnol. 37, 1013–1023. 31406326 10.1038/s41587-019-0198-8 PMC7243676

[73] Paoletti P. Ellis-Davies G.C.R. Mourot A. (2019) Optical control of neuronal ion channels and receptors. Nat. Rev. Neurosci. 20, 514–532. 31289380 10.1038/s41583-019-0197-2 PMC6703956

[74] Schmucker D. Su A.L. Beermann A. Jackle H. Jay D.G. (1994) Chromophore-assisted laser inactivation of patched protein switches cell fate in the larval visual system of *Drosophila*. Proc. Natl. Acad. Sci. U.S.A. 91, 2664–2668. 8146172 10.1073/pnas.91.7.2664 PMC43430

[75] Marek K.W. Davis G.W. (2002) Transgenically encoded protein photoinactivation (FlAsH-FALI): Acute inactivation of synaptotagmin I. Neuron 36, 805–813. 12467585 10.1016/s0896-6273(02)01068-1

[76] Yoshina S. Sakaki K. Yonezumi-Hayashi A. Gengyo-Ando K. Inoue H. Iino Y. (2012) Identification of a novel ADAMTS9/GON-1 function for protein transport from the ER to the Golgi. Mol. Biol. Cell 23, 1728–1741. 22419820 10.1091/mbc.E11-10-0857 PMC3338439

[77] Chuang M. Goncharov A. Wang S. Oegema K. Jin Y. Chisholm A.D. (2014) The microtubule minus-end-binding protein patronin/PTRN-1 is required for axon regeneration in *C. elegans*. Cell Rep. 9, 874–883. 25437544 10.1016/j.celrep.2014.09.054 PMC4250830

[78] Wojtovich A.P. Wei A.Y. Sherman T.A. Foster T.H. Nehrke K. (2016) Chromophore-assisted light inactivation of mitochondrial electron transport chain complex II in *Caenorhabditis elegans*. Sci. Rep. 6, 29695. 27440050 10.1038/srep29695 PMC4954975

[79] Mikuni T. Nishiyama J. Sun Y. Kamasawa N. Yasuda R. (2016) High-throughput, high-resolution mapping of protein localization in mammalian brain by *in vivo* genome editing. Cell 165, 1803–1817. 27180908 10.1016/j.cell.2016.04.044 PMC4912470

[80] Nishiyama J. Mikuni T. Yasuda R. (2017) Virus-mediated genome editing via homology-directed repair in mitotic and postmitotic cells in mammalian brain. Neuron 96, 755–768. e755. 29056297 10.1016/j.neuron.2017.10.004 PMC5691606

[81] Pushpan S.K. Venkatraman S. Anand V.G. Sankar J. Parmeswaran D. Ganesan S. (2002) Porphyrins in photodynamic therapy: A search for ideal photosensitizers. Curr. Med. Chem. Anticancer Agents 2, 187–207. 12678743 10.2174/1568011023354137

[82] Fujimura D. Inagaki F. Okada R. Rosenberg A. Furusawa A. Choyke P.L. (2020) Conjugation ratio, light dose, and pH affect the stability of panitumumab–IR700 for near-infrared photoimmunotherapy. ACS Med. Chem. Lett. 11, 1598–1604. 32832029 10.1021/acsmedchemlett.0c00262 PMC7429969

[83] Mitsunaga M. Ogawa M. Kosaka N. Rosenblum L.T. Choyke P.L. Kobayashi H. (2011) Cancer cell-selective *in vivo* near infrared photoimmunotherapy targeting specific membrane molecules. Nat. Med. 17, 1685–1691. 22057348 10.1038/nm.2554 PMC3233641

[84] Trewin A.J. Berry B.J. Wei A.Y. Bahr L.L. Foster T.H. Wojtovich A.P. (2018) Light-induced oxidant production by fluorescent proteins. Free Radic. Biol. Med. 128, 157–164. 29425690 10.1016/j.freeradbiomed.2018.02.002 PMC6078816

